# Noise Induces Oscillation and Synchronization of the Circadian Neurons

**DOI:** 10.1371/journal.pone.0145360

**Published:** 2015-12-21

**Authors:** Changgui Gu, Jinshan Xu, Jos Rohling, Huijie Yang, Zonghua Liu

**Affiliations:** 1 Business School, University of Shanghai for Science and Technology, Shanghai, P. R. China; 2 College of Computer Science and Technology, Zhejiang University of Technology, Hangzhou, P. R. China; 3 Department of Molecular Cell Biology, Laboratory for Neurophysiology, Leiden University Medical Center, Leiden, The Netherlands; 4 Department of Physics, East China Normal University, Shanghai, P. R. China; University of Lübeck, GERMANY

## Abstract

The principle clock of mammals, named suprachiasmatic nucleus (SCN), coordinates the circadian rhythms of behavioral and physiological activity to the external 24 h light-dark cycle. In the absence of the daily cycle, the SCN acts as an endogenous clock that regulates the ~24h rhythm of activity. Experimental and theoretical studies usually take the light-dark cycle as a main external influence, and often ignore light pollution as an external influence. However, in modern society, the light pollution such as induced by electrical lighting influences the circadian clock. In the present study, we examined the effect of external noise (light pollution) on the collective behavior of coupled circadian oscillators under constant darkness using a Goodwin model. We found that the external noise plays distinct roles in the network behavior of neurons for weak or strong coupling between the neurons. In the case of strong coupling, the noise reduces the synchronization and the period of the SCN network. Interestingly, in the case of weak coupling, the noise induces a circadian rhythm in the SCN network which is absent in noise-free condition. In addition, the noise increases the synchronization and decreases the period of the SCN network. Our findings may shed new light on the impact of the external noise on the collective behavior of SCN neurons.

## Introduction

The primary clock, which is located in the suprachiasmatic nucleus (SCN) in the brain of mammals, regulates the circadian (~24 h) rhythm in physiology and in behavioral activity[1]. The SCN is composed of about twenty thousand self-oscillating neurons, with intrinsic periods ranging roughly from 22 h to 28 h[2]. These non-identical neurons are coupled through neurotransmitters and neuropeptides (e.g. vasoactive intestinal polypeptide, arginine vasopressin and γ-aminobutric acid) to form an SCN network and exhibit collective behavior[3]. On the one hand, the SCN synchronizes bodily rhythms to the external 24 h light-dark cycle (daily cycle); on the other hand, in the absence of the light-dark cycle, the SCN acts as a generator of circadian rhythms and regulates these rhythms throughout our body with an intrinsic period (called free running period) close to but not exactly 24 h[4].

In order to understand the collective behavior of the SCN neuronal population, both experimental and theoretical work have confirmed that the coupling between the SCN neurons pays a pivotal role[5,6,7,8,9,10,11]. The collective behavior of the SCN neuronal network differs if the coupling is either weak or strong[7,9,10]. In the case of weak coupling, the amplitude of the SCN network is damped or reduced and the circadian rhythm of the SCN is lost or weakened. Due to the increase of the coupling strength, the amplitude of the neuronal oscillators is restored, the synchronization between neurons is enhanced and the period of the SCN is increased.

Experimental scientists have found that the collective behavior of the SCN neuronal population is also influenced by external noise[12,13,14]. For example, electrical light pollution shows a role in disrupting the circadian rhythm[13]. Recently, researchers found that external noise (dim light, < 0.2 lux) at night accelerates adjustment to time zone travel[12], and external noise (dim illumination, < 0.005 lux) during dark periods increases the entrainment ability in hamsters[14]. In the absence of an external light-dark cycle, the free running periods of humans have been reported to vary with noise in the experimental environment[15,16,17]. Under carefully controlled lighting conditions (little noise), the free running period is 24.18 h in both young and old people[15]. In a noisy environment, the free running period of the human body temperature rhythm fluctuates, ranging from 24.2 to25.1 hours[15,16].

To the best of our knowledge, no theoretical study has discussed the influence of external noise on the circadian clock in controlled experimental conditions. In the present study, we use coupled Goodwin oscillators to model the SCN network under constant darkness accompanied by external noise. In the Goodwin model, one neuronal oscillator is described by a negative transcriptional-translational feedback loop[7,18,19,20,21]. The neurons are coupled through mean field of coupling to form the SCN network. The external noise (multiplicative noise) originates from variation in external signals to the oscillators, e.g. the fluctuation of the light intensity induced by clouds or light pollution [22,23]. In the rest of this article, we will use the Goodwin model to examine the effect of the interplay between external noise and the coupling strength on network behavior of the SCN, i.e. the oscillation, the synchronization and the free running period of the SCN network.

## Methods

In the Goodwin model, the variables *X*_*i*_, *Y*_*i*_, and *Z*_*i*_ constitute a negative feedback loop in clock cell-*i*, where *X* represents a certain clock gene mRNA, *Y* a clock protein, and *Z* a transcriptional inhibitor. *F* acts as a mean field of transmitter *V* which is produced by *X*, and *g* describes the absorbing ability of the neuron to the mean field *F* i.e. coupling strength[7,24]. The description of the Goodwin model under constant conditions and accompanied by external noise (for example light pollution) is given below:

$$\begin{array}{l} \frac{dX_{i}}{dt}=\alpha_{1}\frac{k_{1}^{n}}{k_{1}^{n}+Z_{i}^{n}}-\alpha_{2}\frac{X_{i}}{k_{2}+X_{i}}+\alpha_{c}\frac{gF}{k_{c}+gF}+X_{i}\zeta_{i}\\ \frac{dY_{i}}{dt}=k_{3}X_{i}-\alpha_{4}\frac{Y_{i}}{k_{4}+Y_{i}}\\ \frac{dZ_{i}}{dt}=k_{5}Y_{i}-\alpha_{6}\frac{Z_{i}}{k_{6}+Z_{i}}\\ \frac{dV_{i}}{dt}=k_{7}X_{i}-\alpha_{8}\frac{V_{i}}{k_{8}+V_{i}}\\ F=\frac{1}{N}\sum_{j=1}^{N}V_{j} \end{array}$$ (1)

where *N* is the total number of neurons and 1 ≤ *i* ≤ *N*. In the present study, the transmitter *V*_*i*_ is taken as a marker of the evolution of oscillator *i* and the mean field *F* is a marker of the evolution of the SCN network. The values of other parameters are set as given in Ref.[24]:

$$\begin{array}{l} \alpha_{1}=6.8355\text{n}M/h,\;k_{1}=2.7266nM,\;n=5.6645,\;\alpha_{2}=8.4297nM/h,\;k_{2}=0.2910nM\\ k_{3}=0.1177/h,\;\alpha_{4}=1.0841nM/h,\;k_{4}=8.1343nM,\;k_{5}=0.3352/h,\;\alpha_{6}=4.6645nM/h\\ k_{6}=9.9849nM,\;k_{7}=0.2282/h,\;\alpha_{8}=3.5216nM/h,\;k_{8}=7.4519nM\\ \alpha_{c}=6.7924nM/h,\;k_{c}=4.8283nM \end{array}$$

**Fig 1:**
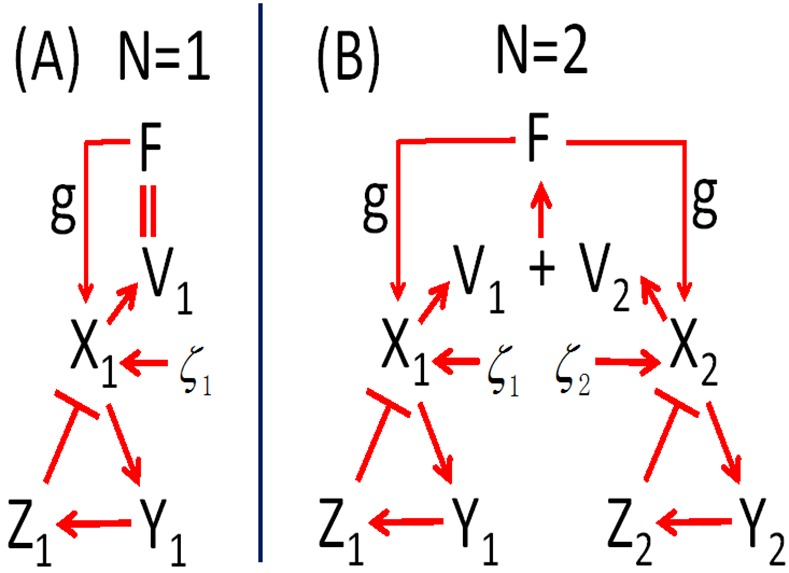
Schematic diagram of the Goodwin model. (A) the number of neuronal oscillators *N* is 1, i.e. the neuronal oscillator is isolated. The clock gene mRNA *X*, clock protein *Y* and transcriptional inhibitor *Z* constitute a negative feedback loop. The transmitter *V* is produced by *X* and then *X* absorbs the mean field *F* which is, in this case, equal to *V*, with the coupling strength (absorbing ability) *g*. *ζ* stands for the external noise. (B) the number of neuronal oscillators in a network, where the number *N* is 2. The mean field *F* is the mean value of the transmitter from these two neurons. We used the first-order Milshtein method[27,28] for numerical simulations of the Goodwin model as presented in Eq 1 with the time increment of 0.001 h. The equations of the Eq 1 are represented as:
$$\begin{array}{l} X_{i}(t+\Delta t)=X_{i}(t)+(\alpha_{1}\frac{k_{1}^{n}}{k_{1}^{n}+Z_{i}^{n}(t)}-\alpha_{2}\frac{X_{i}(t)}{k_{2}+X_{i}(t)}+\alpha_{c}\frac{gF(t)}{k_{c}+gF(t)})\Delta t\\ \;+\frac{D^{2}X_{i}(t)}{2}\Delta t+X_{i}(t)\sqrt{D^{2}\zeta_{i}(t)\Delta t}\\ Y_{i}(t+\Delta t)=Y_{i}(t)+(k_{3}X_{i}(t)-\alpha_{4}\frac{Y_{i}(t)}{k_{4}+Y_{i}(t)})\Delta t\\ Z_{i}(t+\Delta t)=Z_{i}(t)+(k_{5}Y_{i}(t)-\alpha_{6}\frac{Z_{i}(t)}{k_{6}+Z_{i}(t)})\Delta t\\ V_{i}(t+\Delta t)=V_{i}(t)+(k_{7}X_{i}(t)-\alpha_{8}\frac{V_{i}(t)}{k_{8}+V_{i}(t)})\Delta t\\ F(t+\Delta t)=\frac{1}{N}\sum_{j=1}^{N}V_{j}(t) \end{array}$$(2)
where Δ*t* is the time increment. The last two terms of the right side of the first equation in Eq 2 represent the noise terms. The initial 5,000,000 time steps were neglected in order to avoid the influence of transients. The number of oscillators *N* was 500. The initial conditions for each variable were selected randomly from a uniform distribution in the range (0–1) for *X*, *Y*, *Z*, and *V* in the Goodwin model.

Experiments have shown that light triggers the expression of clock genes[25] and thus a light term was added to the first equation of the Eq 1 in some theoretical studies[7,24]. Accordingly, we added the external light noise term *X*_*i*_ *ζ*_*i*_ to the first equation of the Eq 1. We assume that the noise is multiplicative due to the fluctuation of the external signal[23]. *ζ*_*i*_ is the Gaussian white noise, satisfying 〈*ζ*(*t*)〉 = 0, 〈*ζ*_*i*_(*t*)*ζ*_*j*,*j* ≠ *i*_(*t*)〉 = 0 and 〈*ζ*(*t*)*ζ*(*t*')〉 = *D* ^2^ *δ*(*t*−*t*'), where 〈…〉 represents the average over time and *D* is the noise intensity. In the present work, we investigated the effect of the external noise on the SCN network rhythmicity which depends on the synchronization between the neurons. If the SCN neurons are completely out of synchronization, the network rhythmicity is absent. While the SCN neurons are completely in synchronization, the amplitude of the network rhythm is achieved maximally. Consequently, we took the synchronization of the neuronal oscillators and the period of the mean field (SCN network) into account. The period is calculated as in Ref.[26], and the synchronization degree between oscillators is measured over time as[7]:

$$R=\frac{\langle F^{2}\rangle -{\langle F\rangle }^{2}}{\frac{1}{N}\sum_{i=1}^{N}\left(\langle V_{i}^{2}\rangle -{\langle V_{i}\rangle }^{2}\right)}=\frac{Var_{t}(F)}{Mean_{i}(Var_{t}(V_{i}))}$$

where 〈…〉 represents average over time. *R* is 0 for unsynchronized oscillators and 1 for perfect synchronization. In the synchronized state, all the oscillatory *V*_*i*_ have the same period *T* which is equal to the period of the SCN network. As reported in [7,24], the degree *R*, the period *T* as well as the amplitude of the oscillators are governed by the coupling strength *g*. When the coupling strength *g* is larger than 0.8, the oscillators maintain robust synchronization and high amplitude, and when the coupling strength *g* is not larger than 0.8, the oscillators are damped and the amplitude decreases to 0 [24]. In the following sections, let the coupling strength of *g* > 0.8 be strong coupling and the coupling strength of *g* ≤ 0.8 be weak coupling. When the number of neuronal oscillators *N* is equal to 1, the individual neuron is isolated. In the case of strong coupling, the isolated individual neuron shows robust circadian rhythm and is considered to be self-sustaining, and in the case of weak coupling, the isolated neuron is considered to be damped, ultimately leading to amplitude 0. A schematic diagram of the Goodwin model is shown in Fig 1.

We also simulated the effects of the multiplicative noise on the collective behaviors of non-identical oscillators (See S1 File in the Supporting Information). In addition, we studied the influence of the additive (internal) noise on the collective behaviors of neuron oscillators (See S2 File in the Supporting Information).

## Results

### The effect of noise in the case of strong coupling

**Fig 2:**
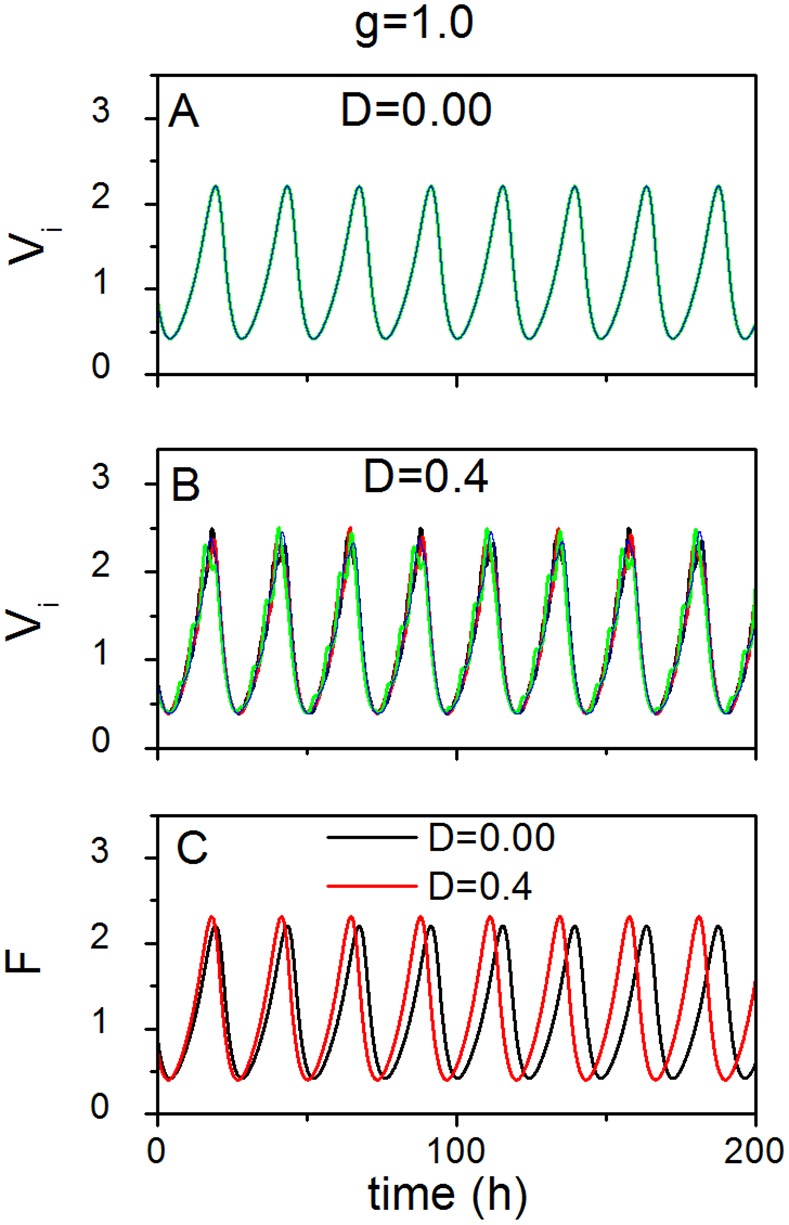
Time series with/without noise in the case of strong coupling. The evolution of four randomly chosen oscillators without noise *D* = 0 (A), and with noise *D* = 0.4 (B). (C) The evolution of the mean field in the case of *D* = 0 and *D* = 0.4 respectively. *g* represents the coupling strength.

Without losing generality, we select *g* = 1.0 as an example of strong coupling. An illustrative example of the effect of noise is shown in Fig 2, when the coupling is strong. In (A), the oscillators maintain perfect synchrony in noise-free conditions (*D* = 0.0). The synchronization degree *R* is slightly reduced when noise intensity increases (*D* = 0.4), and the amplitude of the individual oscillators fluctuates over time (B). In (C), the SCN network (mean field *F*) runs faster in the presence of noise than in the absence of noise. The network amplitude in the presence of noise is slightly larger than in the absence of noise, but in both cases, the amplitude does not dampen over time.

**Fig 3:**
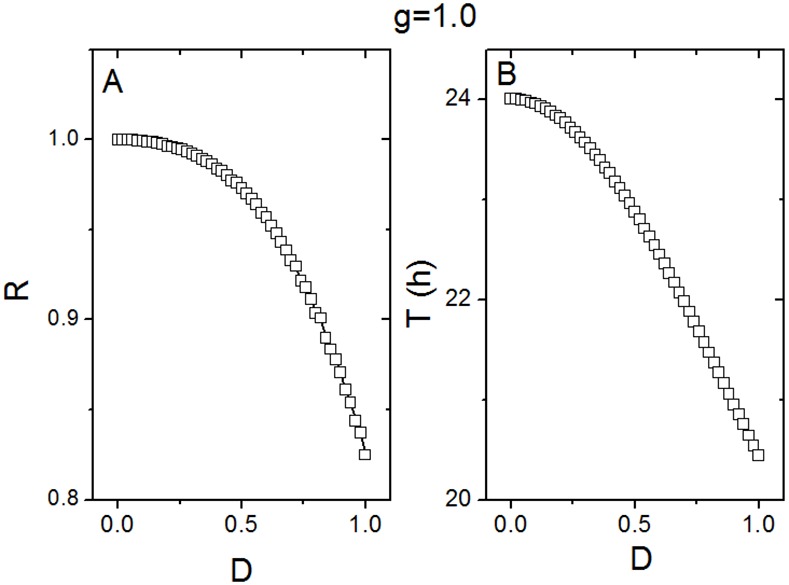
The effect of noise on the collective behavior of the SCN neuronal oscillators in the case of strong coupling. (A) The relationship between the synchronization degree *R* and the noise intensity *D*. (B) The relationship between the period *T* of the SCN population and the noise intensity *D*. *g* represents the coupling strength.

Next, the relationships of the parameters synchronization degree *R* and period *T* to the noise intensity *D* are shown in Fig 3. In (A), the degree *R* monotonically decreases with the increase of the noise intensity *D*. This result confirms the features shown in Fig 1A and 1B. The oscillators show perfect synchrony (*R* = 1) without noise (*D* = 0.0), while the synchrony becomes less (*R* < 1) when the noise increases (*D* > 0.0). In (B), the free running period *T* of the network also monotonically decreases when the noise intensity *D* increases. Note that, if the noise intensity *D* is larger than 1, the variables *X*, *Y*, *Z*, and *V* in Eq 2 achieve negative values. Thus the noise intensity *D* is chosen from 0 to 1. We also simulated the case of *g* = 0.90 (See S4 File in the Supporting Information), and found that the relationships of the degree *R* to the noise intensity *D* and the period *T* to the noise intensity *D* are similar as shown in Fig 3

### The effect of noise in the case of weak coupling

**Fig 4:**
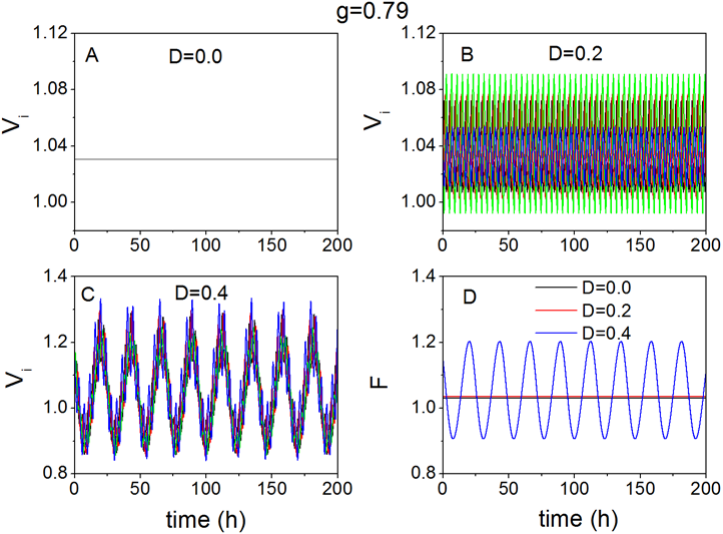
Time series with/without noise in the case of weak coupling. The evolution of four randomly chosen oscillators without noise *D* = 0 (A), with weak noise *D* = 0.2 (B) and with strong noise *D* = 0.4 (C). (D) The evolution of the mean field (network) in the case of *D* = 0, *D* = 0.2 and *D* = 0.4 respectively. *g* represents the coupling strength.

Without losing generality, we select *g* = 0.79 as an example of the weak coupling. When the coupling is weak (*g* = 0.79), the effect of noise on the SCN oscillators and the SCN network is examined in Fig 4. In case there is no noise (*D* = 0.0), the individual neuron shows no oscillation as the amplitude diminishes to zero (A). When the noise is weak (*D* = 0.2), the amplitude of the individual oscillators is recovered, thus the oscillation of the individual neurons shows a rhythm, but the synchronization between the oscillators is lost (B). The oscillators maintain robust synchronization, and exhibit remarkable amplitude with strong noise *D* = 0.4 in (C). In (D), there is no rhythm of the mean field (network) with *D* = 0.0 or *D* = 0.2. If there is no noise, the amplitude of individual oscillators is 0 which causes the absence of rhythms of the mean field (network). In case of weak noise (*D* = 0.2), the individual oscillators are completely out of synchronization, which leads to an amplitude of 0 for the mean field (network). With strong noise (*D* = 0.4), the oscillation of the mean field (network) exhibits a robust circadian rhythm with a fixed amplitude over time.

**Fig 5:**
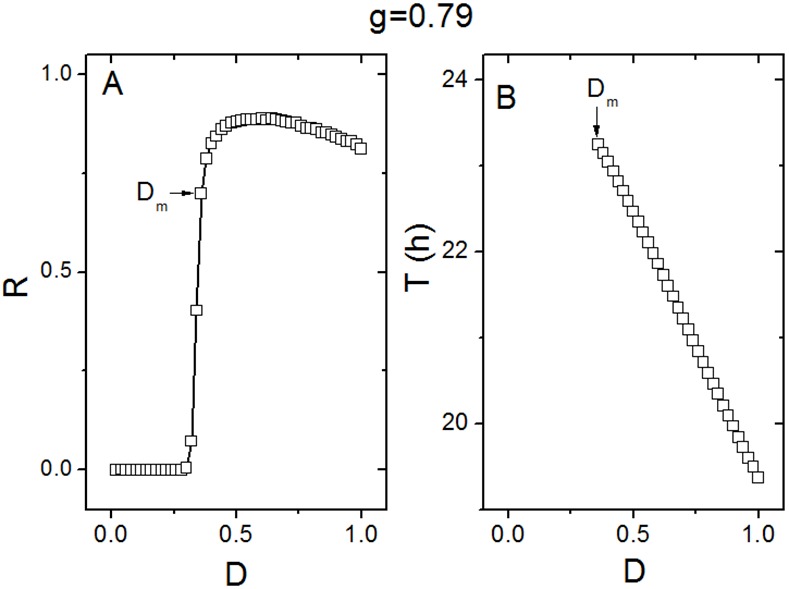
The effect of noise on the collective behavior of the SCN neuronal oscillators in the case of weak coupling. (A) The effect of noise on the synchronization degree *R* between the oscillators. (B) The effect of noise on the period *T* of the SCN network. The minimal noise intensity *D*_*m*_ is 0.36, at which *R* reaches 0.5. *g* represents the coupling strength.

The quantitative relationships of the synchronization degree *R* and the period *T* to the noise intensity *D* are examined respectively in Fig 5A and 5B, for conditions of weak coupling (*g* = 0.79). The relationship of *R* to *D* is not monotonically decreasing, which is different from the results shown in Fig 3. In (A), there are two visible regions, i.e. the left region where *D* < 0.36 corresponds to low synchronization and the right region where *D* ≥ 0.36 corresponds to high synchronization. We defined that *R* ≥ 0.5 represents high synchronization degree and *R* < 0.5 represents low synchronization degree. In this case, *D*_*m*_ = 0.36 is the minimal noise intensity that reaches high synchrony between the oscillators (*R* ≥ 0.5). In the region of *D* < *D*_*m*_, when *D* ≤ 0.30, *R* is almost 0, which means that there is almost no synchrony between the oscillators. When *D* is slightly larger than 0.30, the synchronization degree *R* increases abruptly. In the region of *D* ≥ *D*_*m*_, the synchronization degree *R* is more stable. When *D* is small, it is impossible to calculate the period *T* of the network, because the lack of synchronization leads to amplitude 0 of the network. Accordingly, the period *T* is only calculated for *D* ≥ *D*_*m*_. Qualitatively similar to the results shown in Fig 3(B), the period *T* decreases monotonically with the increase of the noise intensity when *D* ≥ *D*_*m*_ (Fig 5B). We also simulated the case of *g* = 0.75 (See S5 File in the Supporting Information), and found that the relationships of the degree *R* to the noise intensity *D* and the period *T* to the noise intensity *D* are similar as shown in Fig 5.

**Fig 6:**
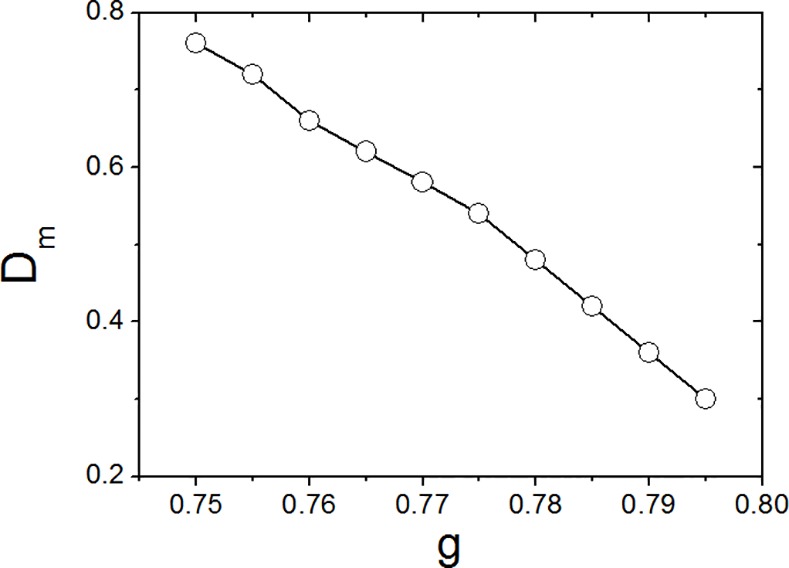
The relationship between the minimal noise intensity *D*_*m*_ and coupling strength g.

In addition to the coupling strength of *g* = 0.75, we studied coupling where strength *g* is smaller than 0.79. Intuitively, if coupling strength *g* becomes smaller, the minimal noise intensity *D*_*m*_ required to maintain high synchrony (*R* ≥ 0.5) and as such maintain a ~24 h rhythm of the SCN network is larger. This intuition is confirmed by Fig 6.

### Analytical results

In order to analytically examine the results from the previous sections and provide explanations to these results, a stability analysis of the fixed point is applied[29]. To perform the analysis it is necessary to find the fixed points and then calculate the eigenvalues of the Jacobian matrix of Eq 2. The fixed points of the difference equations are divided into two types, i.e. the stable one which corresponds to the case where the maximum of the real parts of the eigenvalues of the Jacobian matrix is smaller than 1, and the unstable one which is reflected by the maximal real parts of the eigenvalues being no smaller than 1. The noise terms of the first equation in Eq 2 are $\frac{D^{2}X_{i}(t)}{2}\Delta t$ and $X_{i}(t)\sqrt{D^{2}\zeta_{i}(t)\Delta t}$. Both are determined by the noise intensity *D*, but the latter is also dependent on the random number *ζ*_*i*_(*t*). First, we only considered the first noise term ($\frac{D^{2}X_{i}(t)}{2}\Delta t$), which means that the oscillators are identical. For simplicity, we chose *N* = 1 and focused on the status of fixed points. Letting *X*_*i*_(*t* + Δ*t*) = *X*_*i*_(*t*), *Y*_*i*_(*t* + Δ*t*) = *Y*_*i*_(*t*), *Z*_*i*_(*t* + Δ*t*) = *Z*_*i*_(*t*), and *V*_*i*_(*t* + Δ*t*) = *V*_*i*_(*t*), Eq 2 without the latter noise term can be written as:

$$\begin{array}{l} 0=\alpha_{1}\frac{k_{1}^{n}}{k_{1}^{n}+Z^{n}}-\alpha_{2}\frac{X}{k_{2}+X}+\alpha_{c}\frac{gV}{k_{c}+gV}+\frac{D^{2}X}{2}\\ 0=k_{3}X-\alpha_{4}\frac{Y}{k_{4}+Y}\\ 0=Z+k_{5}Y-\alpha_{6}\frac{Z}{k_{6}+Z}\\ 0=V+k_{7}X-\alpha_{8}\frac{V}{k_{8}+V} \end{array}$$ (3)

**Fig 7:**
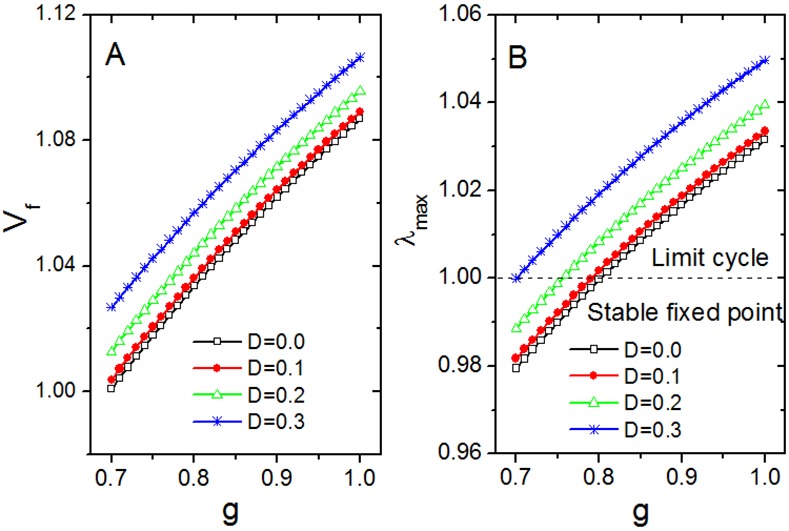
Stability analysis of the fixed points for four noise intensities. (A) The fixed points represented by the marker *V*_*f*_ versus coupling strength *g* for four different values of noise intensity *D*. (B) The maximal real parts *λ*_max_ of the eigenvalues of the Jacobian matrix of the Eq 3 versus coupling strength *g* for four different values of noise intensity *D*. The dashed line of *λ*_max_ = 1 divides the investigated area into two regions, in one of which the fixed points are stable due to *λ*_max_ < 1, and in the other where the fixed points are unstable due to *λ*_max_ ≥ 1 and limit cycles (oscillations) emerge.

Eq 3 is a set of nonlinear equations. We use the numerical method to find the fixed points. The marker *V*_*f*_ is selected to represent the fixed point (*X*_*f*_, *Y*_*f*_, *Z*_*f*_, *V*_*f*_). The relationship between *V*_*f*_ and the noise intensity *D* can be derived from Fig 7(A). *V*_*f*_ increases with the increase of *D* (for each value of *g*). Moreover, *V*_*f*_ increases if the coupling strength *g* increases for all values of *D*.

The Jacobian matrix is shown in Eq 4. The maximal real parts of eigenvalues of the Jacobian matrix are exhibited when (*X*,*Y*,*Z*,*V*) = (*X*_*f*_,*Y*_*f*_,*Z*_*f*_,*V*_*f*_) in Fig 7(B). The dashed line *λ*_max_ = 1 separates the plane into two regions, one region where there is a stable fixed point in which the network population does not show a rhythmic output (*λ*_max_ < 1) and the other region where the network does show rhythmic i.e. limit cycle behavior (*λ*_max_ ≥ 1). In the case of strong coupling *g* = 1.0 or *g* = 0.9, *λ*_max_ > 1 is independent of the noise intensity *D*. Hence, the fixed points are unstable and the limit cycles emerge for any value of *D*. For strong coupling, oscillations in the network are independent from noise.

$$J=\left[\begin{array}{cccc} 1+\frac{D^{2}}{2}-\frac{\alpha_{2}k_{2}}{{\left(k_{2}+x_{a}\right)}^{2}} & 0 & -\frac{n\alpha_{1}k_{1}^{n}z_{a}^{n-1}}{{\left(k_{1}^{n}+z_{\alpha }^{n}\right)}^{2}} & \frac{gk_{c}\alpha_{c}}{{\left(k_{c}+gV\right)}^{2}}]\\ k_{3} & 1-\frac{-\alpha_{4}k_{4}}{{\left(k_{4}+y_{a}\right)}^{2}} & 0 & 0\\ 0 & k_{5} & 1-\frac{\alpha_{6}k_{6}}{{\left(k_{6}+z_{a}\right)}^{2}} & 0\\ k_{7} & 0 & 0 & 1-\frac{-\alpha_{8}k_{8}}{{\left(k_{8}+V\right)}^{2}} \end{array}\right]$$ (4)

In the case of weak coupling strength, there is a break point where *λ*_max_ = 1. For example, for *g* = 0.79 the break point appears for *D* = 0.12 and for *g* = 0.75 this breakpoint happens for *D* = 0.22. When the noise intensity *D* is lower than the break point value (*D* < 0.12 for *g* = 0.79 and *D* < 0.22 for *g* = 0.75), *λ*_max_ < 1 and a stable fixed point emerges i.e. there will be no oscillation in the network as the amplitude is reduced to zero. When the noise intensity *D* is no smaller than the break point value (*D* ≥ 0.12 for *g* = 0.79 and *D* ≥ 0.22 for *g* = 0.75), *λ*_max_ ≥ 1 and limit cycles (oscillations) appear due to the unstable fixed points. Thus, the noise can induce the oscillation from the fixed points when the coupling strength is weak.

After investigating only the first noise term, we now focus on investigating the synchronization degree *R* between the oscillators. The degree *R* is affected by both noise terms of the first equation of Eq 2, which are $\frac{D^{2}X_{i}(t)}{2}\Delta t$ and $X_{i}(t)\sqrt{D^{2}\zeta_{i}(t)\Delta t}$. Without the latter noise term, the *N* oscillators are identical and maintain perfect synchronization. The latter noise term disturbs the synchronization due to the random number *ζ*_*i*_(*t*). Since the former noise term is proportional to *D* ^2^ and the latter noise term is proportional to *D*, when *D* is small (close to 0.0), the latter is dominant and the synchronization is disturbed, and when *D* is large (far away from 0.0), the former plays a more important role and synchronization is enhanced. This explains the sudden increase in synchronization with increasing noise intensity *D* that was shown in Fig 5(A), with the transition where the first noise term becomes more prevalent than the second noise term being around *D*_*m*_.

## Conclusion and Discussion

In the present article, we examined the effect of external (multiplicative) noise on the collective behavior of SCN neuronal oscillators under constant darkness based on the Goodwin model. We observed that noise functions differently for strong coupling and for weak coupling.

**Fig 8:**
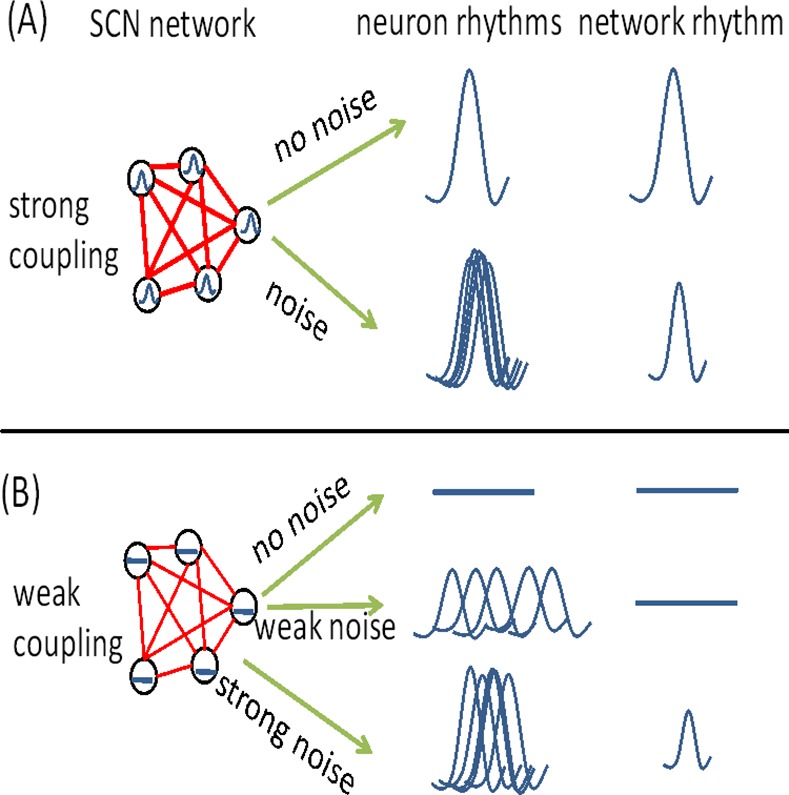
Schematic diagram about the effect of external noise, in the case of strong coupling (A) and in the case of weak coupling (B). The open circle stands for the neuron and the thickness of link represents the coupling strength. Note that, when noise is absent, the neuronal oscillators are perfectly synchronized.

A schematic diagram about the effect of external noise is shown in Fig 8. In case of strong coupling, the individual neurons and the SCN network exhibit a robust circadian rhythm. The noise disturbs the synchronization between the neurons and reduces the period of the SCN network. In case of weak coupling, the amplitude of the individual oscillators went to 0 (stable fixed points) and the rhythm of the SCN network is absent when there is no noise. Application of weak noise (*D* < *D*_*m*_) induces a rhythm in the individual oscillators, but there still are no circadian rhythms at the network level, due to complete desynchronization of the neuronal oscillators. Intriguingly, when strong noise is applied (*D* ≥ *D*_*m*_), a circadian rhythm at the level of both the individual oscillators and the SCN network is observed, and the synchronization between the neurons is remarkable. On the other hand, the effect of noise intensity on the period of the SCN network is similar to the case of strong coupling, i.e. the relationship is monotonically decreasing when the circadian rhythm of the SCN network exists.

In the analytical part, we explained the difference in the noise function between strong coupling and weak coupling. In the case of weak coupling, noise induces oscillations in individual neurons that do not have oscillations without noise. The stable fixed points are influenced by the noise and become unstable which results in oscillatory behavior. Moreover, the synchronization between the neuronal oscillators is determined by the competition between the former noise term and the latter noise term in Eq 2. As the former noise term becomes more important when noise intensity increases, the synchronization degree between the oscillators becomes strong. In the case of strong coupling, the rhythm of the network is the result from the un-stability of the fixed point and is independent of noise. An alternative explanation for the improvement of synchronization by noise could be that the mean of noise terms affects the synchronization[30]. We confirmed this finding (See S5 File in the Supporting Information).

Previous studies have reported that the oscillation of the SCN network is determined by the interplay of the internal (molecular) noise and coupling[31,32,33]. The molecular noise can induce the oscillation of the SCN network even when the individual neurons lack an endogenous circadian rhythm[31]. In the present study, we observed that multiplicative noise can induce the oscillation of the SCN network even when the individual neuronal oscillators are damped, provided there is weak coupling. However, no previous studies have reported the improvement of the synchronization between the neurons by noise. Counter intuitively, we observed that the multiplicative noise not only induces oscillations but also improves the synchronization in case that there is weak coupling.

Whereas the molecular (internal) noise of SCN neurons cannot be easily recorded experimentally as reported in Ref.[31], the external noise is easily determined experimentally. For example, external noise can be induced from the random light pollution under constant darkness. We propose some suggestions for experiments based on our findings. Firstly, in both cases of weak coupling and strong coupling, the period of the SCN network decreases with the increase of the noise intensity. Thus, the environmental noise brought about by the fluctuation in light pollution may reduce the free running period of the animals under constant darkness.

Secondly, the external noise recovers the amplitude and synchronization when the amplitude of individual neuronal oscillators decreases to 0. In the aging human being, circadian rhythms are weakened, as a result from reduced coupling and diminished amplitude of individual neurons[34]. We suggest that application of external noise, e.g. evening light, to elderly human beings may strengthen their circadian rhythms.

Finally, in the present study, the effect of external noise on the ability of the SCN to external zeitgebers (such as the light-dark cycle) is not considered. People usually thought that external noise disturbs the circadian rhythms. On the contrary, the work in Ref.[12] found that external noise accelerates the adjustment of the SCN to a new time zone. In future, the role of the external noise in entrainment should be examined.

## Supporting Information

S1 File The effect of external noise on the collective behaviors of non-identical oscillators. (PDF)

S2 File The effect of internal noise on the collective behaviors of the SCN neuron oscillators. (PDF)

S3 File The effect of external noise in the case of strong coupling *g* = 0.9. (PDF)

S4 File The effect of external noise in the case of weak coupling *g* = 0.75. (PDF)

S5 File The relationship between the mean of the noise terms and the synchronization. (PDF)
