# Supplementary material for: Noise Induces Oscillation and Synchronization of the Circadian Neurons
Source: PLoS One. 2015 Dec 21;10(12):e0145360. doi: 10.1371/journal.pone.0145360 (PMC4687094; doi:10.1371/journal.pone.0145360)
Supplement: S1 File — (PDF) [file pone.0145360.s001.pdf]

The effect of external noise on the collective behaviors of non-identical oscillators

The Goodwin model composed of non-identical oscillators read as:

$$
\begin{aligned}
\frac{dX_i}{dt} &= \sigma_i\left(\alpha_1 \frac{k_1^n}{k_1^n + Z_i^n} - \alpha_2 \frac{X_i}{k_2 + X_i} + \alpha_c \frac{gF}{k_c + gF}\right) + X_i\zeta_i \\
\frac{dY_i}{dt} &= \sigma_i\left(k_3 X_i - \alpha_4 \frac{Y_i}{k_4 + Y_i}\right) \\
\frac{dZ_i}{dt} &= \sigma_i\left(k_5 Y_i - \alpha_6 \frac{Z_i}{k_6 + Z_i}\right) \\
\frac{dV_i}{dt} &= \sigma_i\left(k_7 X_i - \alpha_8 \frac{V_i}{k_8 + V_i}\right) \\
F &= \frac{1}{N}\sum_{j=1}^{N} V_j
\end{aligned}
\qquad \text{(S1)}
$$

where σ_i ensures that the neuronal oscillators are non-identical, and satisfy a normal distribution with mean 1 and the standard deviation η. If σ_i is larger, the oscillator i runs faster. In the previous studies[1,2], η is selected as $\eta = 0.05$. The other parameters are the same as the main text.

The effects of external noise on the synchronization degree R and the network period T are shown in Fig S1. The effects are similar as shown in Fig 3 and Fig 5.

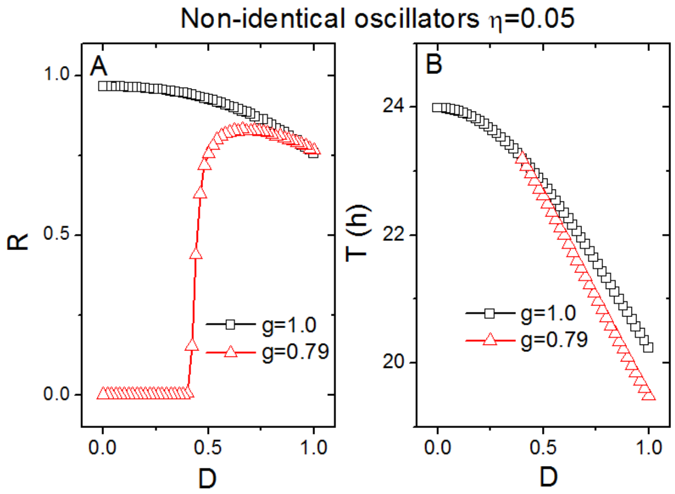

Fig S1. The effect of external noise on the collective behavior of non-identical neuron oscillators. (A) The relationship between the synchronization degree R and the noise intensity D. (B) The relationship between the period of the SCN network T and the noise intensity D. g represents the coupling strength.

Reference:

1. **Gonze D, Bernard S, Waltermann C, Kramer A, Herzel H (2005) Spontaneous synchronization of coupled circadian oscillators. Biophys J 89: 120-129.**

2. **Locke JC, Westermark PO, Kramer A, Herzel H (2008) Global parameter search reveals design principles of the mammalian circadian clock. BMC Syst Biol 2: 22.**
