# Supplementary material for: Noise Induces Oscillation and Synchronization of the Circadian Neurons
Source: PLoS One. 2015 Dec 21;10(12):e0145360. doi: 10.1371/journal.pone.0145360 (PMC4687094; doi:10.1371/journal.pone.0145360)
Supplement: S2 File — (PDF) [file pone.0145360.s002.pdf]

The effect of internal noise on the collective behavior of the SCN neuronal oscillators

The Goodwin model with internal (additive) noise is read as:

$$\begin{aligned}
\frac{dX_i}{dt} &= \alpha_1 \frac{k_1^n}{k_1^n + Z_i^n} - \alpha_2 \frac{X_i}{k_2 + X_i} + \alpha_c \frac{gF}{k_c + gF} + \varsigma_i + X_i \zeta_i \\
\frac{dY_i}{dt} &= k_3 X_i - \alpha_4 \frac{Y_i}{k_4 + Y_i} \\
\frac{dZ_i}{dt} &= k_5 Y_i - \alpha_6 \frac{Z_i}{k_6 + Z_i} \\
\frac{dV_i}{dt} &= k_7 X_i - \alpha_8 \frac{V_i}{k_8 + V_i} \\
F &= \frac{1}{N} \sum_{j=1}^{N} V_j
\end{aligned} \qquad \text{(S2)}$$

where ς_i is the additive Gaussian white noise. ς_i satisfies $\langle \varsigma(t) \rangle = 0$ and $\langle \varsigma(t)\varsigma(t') \rangle = E^2 \delta(t-t')$, where $\langle ... \rangle$ represents average over time and E the noise intensity. The other parameter values are the same as in the main text. For numerical simulation, we used the first-order Milshtein method as in the main text.

$$\begin{aligned}
X_i(t+\Delta t) &= X_i(t) + (\alpha_1 \frac{k_1^n}{k_1^n + Z_i^n(t)} - \alpha_2 \frac{X_i(t)}{k_2 + X_i(t)} + \alpha_c \frac{gF(t)}{k_c + gF(t)})\Delta t \\
&\quad + \sqrt{E^2 \varsigma_i(t) \Delta t} + \frac{D^2 X_i(t)}{2} \Delta t + X_i(t) \sqrt{D^2 \zeta_i(t) \Delta t} \\
Y_i(t+\Delta t) &= Y_i(t) + (k_3 X_i(t) - \alpha_4 \frac{Y_i(t)}{k_4 + Y_i(t)})\Delta t \\
Z_i(t+\Delta t) &= Z_i(t) + (k_5 Y_i(t) - \alpha_6 \frac{Z_i(t)}{k_6 + Z_i(t)})\Delta t \\
V_i(t+\Delta t) &= V_i(t) + (k_7 X_i(t) - \alpha_8 \frac{V_i(t)}{k_8 + V_i(t)})\Delta t \\
F(t+\Delta t) &= \frac{1}{N} \sum_{j=1}^{N} V_j(t)
\end{aligned} \qquad \text{(S3)}$$

The effects of internal noise on the collective behaviors of the SCN neuronal

oscillators are show in Fig S2. It shows that the synchronization degree R decreases with the increase of additive noise intensity E (Panel A), and the network period T decreases with the increase of E (Panel C), for multiplicative noise intensity D=0.0, 0.1, 0.4 and 1.0, provided that the coupling is strong with g=1.0. When the coupling is weak with g=0.79, the additive noise cannot induce the oscillation of the SCN network because the synchronization degree R is zero for multiplicative noise intensities D=0.0 and 0.1.The relationship of synchronization degree R to the additive noise strength E is more complicated for multiplicative noise intensities D=0.4 and 1.0 (Panel B). In Panel B, the relationship is not monotonous and there is a peak of R for D=0.4. For D=1.0 the relationship is negative. The period T is decreased with the increase of E for multiplicative noise intensities D=0.4 and D=1.0 (Panel D). The period T is absent for noise intensities of D=0.0 and D=0.1, because the neuronal oscillators are all out of synchronization.

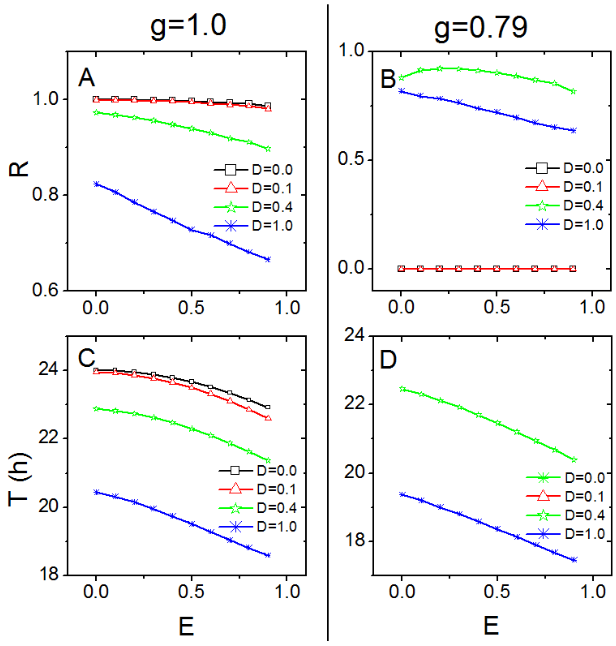

Fig S2. The effect of internal noise on the collective behavior of the SCN neuron oscillators. (A) and (B) The relationship between the synchronization degree R and

the additive noise intensity E. (C) and (B) The relationship between the period of the SCN network T and the additive noise intensity E. g represents the coupling strength, and D stands for the multiplicative noise intensity.
