# Supplementary material for: Noise Induces Oscillation and Synchronization of the Circadian Neurons
Source: PLoS One. 2015 Dec 21;10(12):e0145360. doi: 10.1371/journal.pone.0145360 (PMC4687094; doi:10.1371/journal.pone.0145360)
Supplement: S3 File — (PDF) [file pone.0145360.s003.pdf]

The effect of external noise in the case of strong coupling $g = 0.9$

In the case of strong coupling $g = 0.9$, the synchronization degree R decreases with the increase of the noise intensity D, and the period T decreases with the increase of the noise intensity D (Fig S3). This finding is consistent with shown in Fig 3.

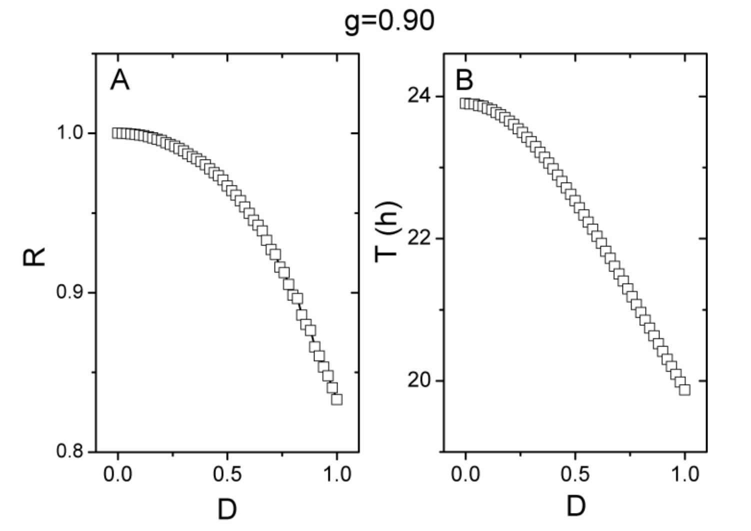

Fig S3. The effect of external noise on the synchronization and the period of the SCN neuronal oscillators in the case of strong coupling $g = 0.9$. (A) The relationship between the synchronization degree R and the noise intensity D. (B) The relationship between the period of the SCN network T and the noise intensity D.
