# Supplementary material for: Noise Induces Oscillation and Synchronization of the Circadian Neurons
Source: PLoS One. 2015 Dec 21;10(12):e0145360. doi: 10.1371/journal.pone.0145360 (PMC4687094; doi:10.1371/journal.pone.0145360)
Supplement: S4 File — (PDF) [file pone.0145360.s004.pdf]

The effect of noise in the case of strong coupling $g = 0.75$

In the case of strong coupling $g = 0.75$, there is a break point $D_m = 0.76$. When $D < D_m$, the synchronization degree R is close to 0 and the calculation of the period T is not accessible. When $D \geq D_m$, the synchronization degree R increases with the increase of the noise intensity D, and the period T is decreased with the increase of the noise intensity D (Fig S4). This finding is consistent with shown in Fig 5.

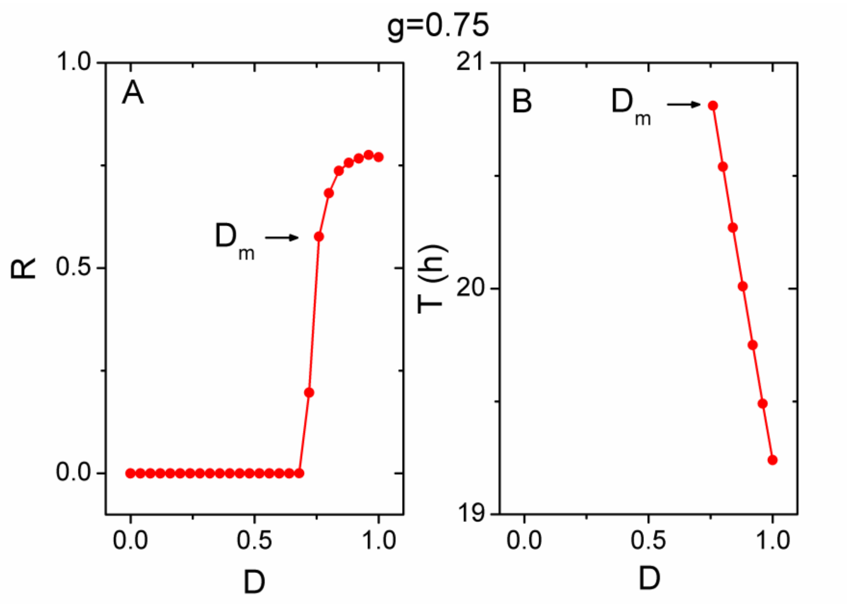

Fig S4. The effect of external noise on the synchronization and the period of the SCN neuron oscillators in the case of weak coupling $g = 0.75$. (A) The relationship between the synchronization degree R and the noise intensity D. (B) The relationship between the period T of the SCN network and the noise intensity D.
