# Supplementary material for: Noise Induces Oscillation and Synchronization of the Circadian Neurons
Source: PLoS One. 2015 Dec 21;10(12):e0145360. doi: 10.1371/journal.pone.0145360 (PMC4687094; doi:10.1371/journal.pone.0145360)
Supplement: S5 File — (PDF) [file pone.0145360.s005.pdf]

The relationship between the mean of the noise terms and the synchronization

The mean of the multiplicative noise terms in the Eq.(2) is defined as:

$$\left\langle \frac{1}{N}\sum_{i=1}^{N}\frac{D^2 X_i(t)}{2}\Delta t + X_i(t)\sqrt{D^2\zeta_i(t)\Delta t} \right\rangle$$

where $\langle ... \rangle$ denotes the average over time. The mean of the external noise terms is shown in Fig **S5.** It's clear that the mean increases with the noise intensity D and is no smaller than 0, in both the cases of g=1.0 and g=0.79.

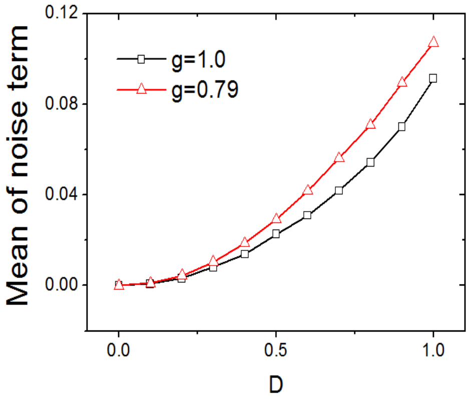

Fig S5. The dependence of the mean of noise terms on the external noise intensity D. g represents the coupling strength.

In Ref.[1], Herzel found that the mean of the noise terms has an effect on the synchrony, i.e. with the increase of the mean, the synchrony is increased in logistic map. Moreover, he found that adding a positive constant also improves the synchronization. Thus, the mean of the noise terms can be presented by a constant. Following his method, we also add a constant to the Goodwin model:

$$\frac{dX_i}{dt} = \alpha_1 \frac{k_1^n}{k_1^n + Z_i^n} - \alpha_2 \frac{X_i}{k_2 + X_i} + \alpha_c \frac{gF}{k_c + gF} + C$$
$$\frac{dY_i}{dt} = k_3 X_i - \alpha_4 \frac{Y_i}{k_4 + Y_i}$$
$$\frac{dZ_i}{dt} = k_5 Y_i - \alpha_6 \frac{Z_i}{k_6 + Z_i} \quad \text{(S4)}$$
$$\frac{dV_i}{dt} = k_7 X_i - \alpha_8 \frac{V_i}{k_8 + V_i}$$
$$F = \frac{1}{N}\sum_{j=1}^{N} V_j$$

where C is a constant. Because the mean of the noise terms is larger than 0 (Fig S5), C is positive. The other parameter values are the same as in the main text.

The effects of the constant C (Fig S6) are similar to the effects of the external noise shown in Fig 3 and Fig 5. Hence the mean of the noise represented by the constant C plays a role in the synchronization.

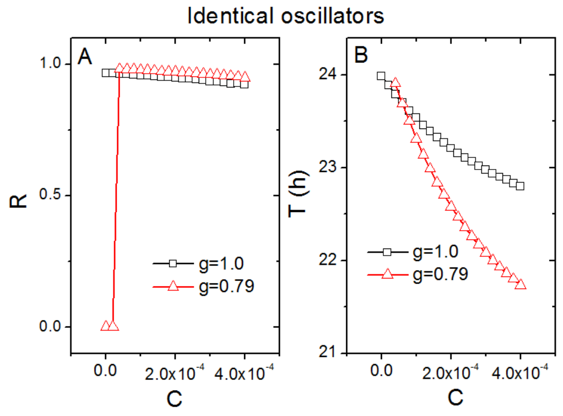

Fig S6. The relationship of synchronization degree R and period T to the additive constant C. g represents the coupling strength.

Reference

1. Herzel H, Freund J (1995) Chaos, noise, and synchronization reconsidered. Phys Rev E Stat Phys Plasmas Fluids Relat Interdiscip Topics 52: 3238-3241.
